# MicroRNAs in Nonalcoholic Fatty Liver Disease

**DOI:** 10.3390/jcm4121953

**Published:** 2015-12-04

**Authors:** György Baffy

**Affiliations:** Department of Medicine, VA Boston Healthcare System and Brigham and Women’s Hospital, Harvard Medical School, 150 S. Huntington Ave., Room 6A-46, Boston, MA 02130, USA; gbaffy@bwh.harvard.edu; Tel.: +1-857-364-4327; Fax: +1-857-364-4179

**Keywords:** nonalcoholic fatty liver disease, steatohepatitis, hepatocellular carcinoma, miRNA, circulating miRNA, antagomir, differential expression, transcriptome, predicted target genes

## Abstract

Nonalcoholic fatty liver disease (NAFLD) has become the most common liver disorder. Strongly linked to obesity and diabetes, NAFLD has the characteristics of complex diseases with substantial heterogeneity. Accordingly, our ability to predict the risk of advanced NAFLD and provide efficient treatment may improve by a better understanding of the relationship between genotype and phenotype. MicroRNAs (miRNAs) play a major role in the fine-tuning of gene expression and they have recently emerged as novel biomarkers and therapeutic tools in the management of NAFLD. These short non-coding RNA sequences act by partial repression or degradation of targeted mRNAs. Deregulation of miRNAs has been associated with different stages of NAFLD, while their biological role in the pathogenesis remains to be fully understood. Systems biology analyses based on predicted target genes have associated hepatic miRNAs with molecular pathways involved in NAFLD progression such as cholesterol and lipid metabolism, insulin signaling, oxidative stress, inflammation, and pathways of cell survival and proliferation. Moreover, circulating miRNAs have been identified as promising noninvasive biomarkers of NAFLD and linked to disease severity. This rapidly growing field is likely to result in major advances in the pathomechanism, prognostication, and treatment of NAFLD.

## 1. Introduction

Nonalcoholic fatty liver disease (NAFLD) is increasingly common in developed societies, affecting between 20% and 40% of the adult population [1,2]. Initially described as abnormal hepatic fat accumulation in the absence of viral, toxic, or genetic causes of liver disease [3], NAFLD is an important component of the metabolic syndrome, associated with visceral obesity, insulin resistance, hyperlipidemia, and endothelial dysfunction [4]. While NAFLD most often presents as steatosis, 20% to 25% of all NAFLD cases are recognized as nonalcoholic steatohepatitis (NASH), displaying a complex pathology that includes hepatocellular injury, inflammation, and a varying degree of liver fibrosis [5,6]. Moreover, NASH evolves into cirrhosis at a rate of 10% to 20% over 10 years and may culminate in major complications such as portal hypertension, liver failure, and hepatocellular carcinoma (HCC) [2,7,8].

Similar to other complex diseases, the pathogenesis and natural history of NAFLD appear to be determined by a rich interplay between genes, gene products, and environmental factors [9,10]. Thanks to recent advances in molecular genetics and systems biology, our understanding of the genotype–phenotype associations in complex diseases has substantially improved [11,12]. For instance, genome-wide association studies (GWAS) have identified a number of single nucleotide polymorphisms (SNPs), which are typically present in 5% or more of the population, with a potential role in wide-ranging disease outcomes including the development and progression of NAFLD [10,11]. However, less than 10% of genetic variance is explained by these common variants and it is possible that much of the phenotypic differences result from rare combinations of common genetic variants [13,14]. This latter notion heavily accounts for the role of gene–environment interactions and may explain why we have failed to define NAFLD heterogeneity at the genomic level.

While the effects of environment do not necessarily have an impact on the genome, epigenetic modulation of gene expression may occur in response to external factors and manifest in three major forms: (i) modification of the DNA nucleotides (e.g., methylation); (ii) alteration of the DNA-binding histone proteins that determine DNA packing and accessibility; and (iii) regulation of transcription by altering mRNA stability and activity due to specific binding of small RNA molecules such as microRNAs [15]. MicroRNAs (miRNAs) represent a fundamental biological mechanism that regulates gene–environment interactions and provides novel insights into the development and manifestation of complex diseases [16,17]. The role of miRNAs as pathogenic factors, risk predictors, and therapeutic targets in NAFLD is the subject of this review.

## 2. A Brief Overview of the miRNAs

MiRNAs are short endogenous RNA sequences consisting of ~22 nucleotides that regulate gene expression by partial repression or degradation of targeted mRNAs as an evolutionarily conserved molecular mechanism to modulate protein synthesis [16]. Currently, there are more than 2000 known miRNAs encoded in various intergenic, intronic, or exonic sequences of our genome and it is estimated that miRNAs may directly target up to 60% of all human genes [18,19]. The biogenesis of miRNAs has been extensively reviewed elsewhere [20]. Briefly, miRNA synthesis begins with the transcription of primary miRNAs (pri-miRNAs) by the RNA polymerase II in the nucleus. These RNA molecules contain several hairpin structures that are subsequently cleaved into precursor miRNAs (pre-miRNAs) by the catalytic RNase III domain of Drosha within a “microprocessor” complex. Pre-miRNAs are further processed by Dicer, a cytosolic RNase III, resulting in the mature miRNA/miRNA* duplex. While the passenger strand (miRNA*) is usually degraded, the strand of choice (miRNA) is incorporated into the RNA-induced silencing complex (RISC) where it interacts with the complementary mRNA. A member of the Argonaute (Ago) protein family, which is a key component of RISC, usually guides this interaction. A number of exceptions to this simplified biogenesis have been recognized [21].

The primary function of miRNAs in mammals is to provide transcriptional fine-tuning rather than an all-out silencing of the targeted genes (which is a protective mechanism during RNA interference directed against exogenous genetic material) [16,17]. Modification of translational activity by miRNA binding occurs if miRNA/mRNA complementarity remains partial beyond the 6–8 nucleotides at the 5′ end of the miRNA (“seed region”), while perfect base pairing initiates degradation of mRNA (which usually occurs in plants) [20,21]. Genetic polymorphism or rare mutations affecting complementarity within the short stretch of the seed region may dramatically change miRNA affinity and function, providing one explanation for individual susceptibility to epigenetic modifications [17]. Further complexity of miRNA biology is derived from the fact that miRNA-coding genes themselves are subject to similar regulatory mechanisms as the protein-coding genes [17,20]. These mechanisms include DNA methylation of miRNA-encoding genes as well as RNA editing and other forms of posttranscriptional modification that may alter miRNA stability and degradation [22,23]. MiRNAs may also create feedback and feed-forward loops by targeting the transcription of their own transcription factors [20,22,23].

Many miRNAs simultaneously target a large number of mRNAs and more than one miRNA can converge onto a single transcript [16,23]. This combinatorial targeting provides miRNAs with an extensive regulatory capacity and a profound impact on health and disease [16,17]. The complexity of miRNAs involves cooperative and antagonistic mechanisms and substantial inconsistency exists among experimental findings and human observations. Few studies have been able to replicate specific findings on different miRNA expression in different conditions, which is an important challenge at this relatively early stage of miRNA research.

## 3. Aberrant miRNA Profiles in Experimental and Human NAFLD

MicroRNAs have recently emerged as novel biomarkers and potential therapeutic targets in the management of NAFLD. Differential miRNA expression has identified a number of miRNAs with increased or decreased abundance associated with human NAFLD or experimental NAFLD induced by dietary or genetic manipulations [22,24,25,26,27]. By virtue of their ability to modulate multiple metabolic and signaling pathways, miRNAs appear to be involved in all stages of NAFLD. Thus, deregulation of miRNAs has been associated with altered lipid and glucose metabolism, oxidative stress, inflammation, and pathways of hepatocellular survival and proliferation [22,24,25,27]. A notable limitation of these studies is that while global miRNA sequencing is increasingly performed, most of currently available reports have been based on microarrays with a limited set of probes and cannot account for all miRNAs potentially involved in a given experimental or observational paradigm.

Diet-induced obesity in mice results in the differential expression of 6% of total miRNAs [24,28]. High-fat diet administered to rats leads to differentially expressed miRNAs including upregulation of miR-146, miR-152, and miR-200 family members with predicted target genes regulating ion and protein transport, cell adhesion, and migration [29]. Importantly, these changes can be similarly observed in human hepatocytes and immortalized liver cell lines exposed to various fatty acids and pro-inflammatory cytokines [29]. Human observations provide additional evidence for aberrant miRNAs in obesity, insulin resistance, diabetes, and NAFLD [30]. In one of the earliest observations in humans, hepatic miRNA profiles of subjects with NASH and the metabolic syndrome by using a microarray of 474 human miRNAs were compared to healthy controls and 46 differentially expressed miRNA species were identified of which 23 were upregulated and 23 were downregulated [31]. Predicted targets of these miRNAs included genes that regulate lipid metabolism, inflammation, oxidative stress, and apoptosis. Intriguingly, however, individual histological features of NAFLD severity showed no correlations with changes in the expression level of these miRNAs [31].

## 4. Specific miRNAs Associated with the Progression of NAFLD

**Figure 1:**
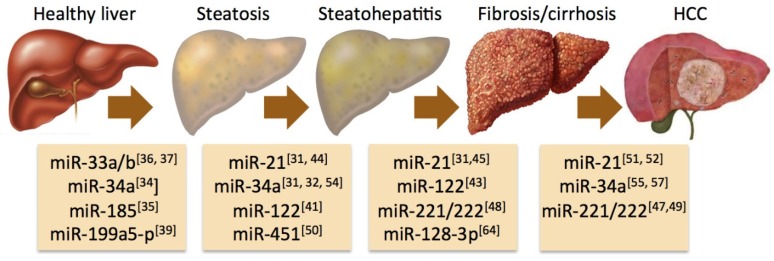
Implication of microRNAs in key transitions of the pathogenesis of nonalcoholic fatty liver disease (NAFLD). Schematic illustration of selected miRNAs shown to have an impact on the natural history of NAFLD with relevant references cited in the review. Please see specific details in the main text.

Several miRNAs have been identified to play key roles in the development of steatosis and its progression to steatohepatitis, fibrosis, cirrhosis, and hepatocellular carcinoma (Figure 1). One of the most lipid-responsive miRNAs in the liver is miR-34a, which is heavily upregulated in mice kept on high-fat diet and its expression levels in humans correlate with the severity of NASH [31,32]. Overexpression of miR-34a results in hepatocellular apoptosis [32]. A major target of miR-34a is the NAD-dependent deacetylase Sirtuin-1 (SIRT1), which has a key role in energy homeostasis by activating pivotal transcription factors such as peroxisome proliferator-activated receptor-alpha (PPARα) and liver X receptor (LXR), while it has an inhibitory effect on PPAR-gamma coactivator-1alpha (PGC-1α), sterol regulatory element-binding protein 1c (SREBP1c), and farnesoid X receptor (FXR) [33]. Silencing of miR-34a restores the expression of SIRT1 and PPARα, resulting in activation of the metabolic sensor AMP-activated protein kinase (AMPK) and the activation of various PPARα target genes, suggesting a fundamental role of miR-34a in the deregulation of lipid metabolism associated with NAFLD [34].

In a recent study, Wang and colleagues examined how loss or gain of miR-185 may affect lipid metabolism and insulin sensitivity in mice fed with high-fat diet and in HepG2 cells exposed to palmitate [35]. They observed significant downregulation on miR-185 in both experimental models, indicating concomitantly increased expression of key genes involved in the regulation of *de novo* lipogenesis and cholesterol synthesis, such as fatty acid synthase (FAS), 3-hydroxy-3-methyl-glutaryl-CoA reductase (HMGCR), SREBP1c, and SREBP2. By contrast, these authors found that overexpression of miR-185 resulted in increased insulin receptor substrate-2 (IRS-2) expression, improved insulin sensitivity and reduced steatosis [35]. Another prominent miRNA involved in the positive regulation of cholesterol and fatty acid biosynthesis is miR-33a/b [36,37]. Since inhibition of miR-33a/b enhances fatty acid oxidation and insulin signaling, it is a potential molecular target in the management of metabolic syndrome [38]. Moreover, miR-199a-5p has been identified as another inhibitor of fatty acid oxidation with a potential amplification loop involved in the mechanism of action since miR-199a-5p expression is increased in human liver cell lines exposed to free fatty acids [39]. This effect seems to involve diminished caveolin-1 (CAV1) and PPARα expression, while suppression of miR199a-5p results in increased ATP and mitochondrial DNA contents consistent with improved cellular energy metabolism [39].

By far the most abundant miRNA in the liver is miR-122, comprising 70% of total miRNAs expressed in this tissue [22,25]. This abundant miRNA species has a key role in the epigenetic regulation of gene expression related to liver health. The predicted targets of miR-122 include genes regulating cholesterol and lipid metabolism, proteasomal protein degradation, cell adhesion and extracellular matrix biology [40]. Consequently, miR-122 appears to be involved in several transitions during the progression of NAFLD and modulation of miR-122 expression can recapitulate many of the changes seen in the natural history of NAFLD. Mice deficient for miR-122 develop normally but will rapidly progress into steatohepatitis [41]. The importance of miR-122 is further supported by studies in which experimental NAFLD is induced by chemical and dietary interventions and accompanied by relative deficiency of miR-122. Thus, miR-122 expression was substantially reduced in the liver of mice fed with a methionine-choline-deficient (MCD) [42]. In a similar experimental paradigm, relative absence of hepatic miR-122 after 8 weeks of MCD diet inducing maximum level of fibrosis was accompanied by increased activation of nuclear factor kappaB (NF-κB) and upregulation of mitogen-activated protein kinase kinase kinase 3 (MAP3K3), hypoxia inducible factor-1 alpha (HIF-1α), and vimentin, supporting a pro-fibrogenic role of miR-122 [43].

In addition to miR-122, several miRNAs have been associated with the pathogenesis of NAFLD. Cheung and colleagues found that miR-21 is heavily upregulated in the liver of patients with steatohepatitis and additional studies confirmed that NAFLD is associated with hepatic miR-21 abundance [31,44]. Recent work on the experimental NASH induced in mice deficient for the low-density lipoprotein (LDL)-receptor by high-fat diet indicates that miR-21 is primarily expressed in liver inflammatory and biliary cells and administration of antagomir-21 can strongly diminish hepatic miR-21 expression in association with reduced hepatocellular injury, inflammation, and fibrosis [45]. The same group corroborated these findings in miR-21 deficient mice fed a methionine/choline-deficient diet and identified PPARα as a major target on miR-21 action in NAFLD [45].

There is evidence that fatty acids regulate the expression of additional miRNAs that in return contribute to the progression of NAFLD beyond steatosis and steatohepatitis. The miR-221/222 family is upregulated in genetically induced obesity of the *ob*/*ob* mice and increased levels have also been observed in the livers of NAFLD patients [46,47]. These changes appear to correlate with hepatic stellate cell activation and the severity of liver fibrosis [48]. In addition, increased expression of miR-221/222 has been associated with early stages of NAFLD-related HCC in mice, in line with observations that their targets include genes involved in cell cycle control (p27) and tumor suppression (PTEN) [27,47,49]. The gene of miR-451 is also responsive to fatty acids since lesser amounts of hepatic miR-451 have been detected in human NASH, in HepG2 cells treated with palmitate, and in mice given high-fat diet [50]. Deficiency in miR-451 leads to increased activation of interleukin-8 (IL-8), tumor necrosis factor-alpha (TNFα), and NF-κB with the promotion of oncogenesis, while experimental miR-451 overexpression inhibits these pathways [50].

Recent work indicates a more complex role for miR-21 in the pathogenesis of NAFLD, describing increased expression of miR-21 in HepG2 cells incubated with fatty acids and in the liver of mice given high-fat diet [51]. This study identified HMG-box transcription factor 1 (HPB1), a transcriptional activator of the tumor suppressor p53, as a major target of miR-21. Knockdown of miR-21 in these experiments restored the expression of HPB1 and p53 while downregulated SREBP1c, pointing to a novel pathway by which miR-21 may affect the function of p53 regulating lipogenesis and cancer development in obesity-induced NAFLD [51]. Additional evidence for a link between miR-21 and hepatocarcinogenesis was gained from studies in which unsaturated fatty acids inhibited PTEN expression in hepatocytes by up-regulating miR-21 via the mammalian target of rapamycin (mTOR)/NF-κB pathway [52].

An additional and intriguing connection has recently been uncovered between the tumor suppressor p53 protein, miR-34a, SIRT1, and steatosis. Since p53 is a key activator of the pro-apoptotic miR-34a gene [53], it diminishes the effect of SIRT1 and promotes hepatic fat accumulation. While proof-of-concept studies indicate that chemical inhibition of p53 by pifithrin attenuates steatosis and associated lipotoxicity liver damage in mice [54], human applicability and safety of this approach remains unclear. Other miR-34a targets with potential role in the progression of NAFLD include genes involved in the Wnt pathway and in endothelial-mesenchymal transition, implicating miR-34a in the regulation of cancer stem cell plasticity [55]. It is well known that conjugated bile acids, such as deoxycholic acid (DCA), induce apoptosis in hepatocytes by activating death receptors [56]. According to a recent report, DCA enhances the miR-34a/SIRT1/p53 pathway of hepatocellular apoptosis by activating p53 with the involvement of c-Jun N-terminal kinase (JNK) 1 and c-Jun, providing further evidence for amplification loops in this process and potentially identifying novel pharmacological targets [57].

## 5. Circulating miRNAs as a Diagnostic Tool in NAFLD

There is increasing evidence that significant amounts of miRNAs can be detected in various bodily fluids including serum and saliva [58,59]. While extracellular RNAs are typically short-lived due to an almost ubiquitous RNase activity, short sequences of circulating endogenous miRNAs have proved to be remarkably stable, indicating their potential utility as biomarkers in health and disease [58,59]. Analysis of circulating miRNAs indicates that there are different forms of miRNA-carrying particles in the bloodstream [22,59]. Circulating miRNAs may be found in a non-membrane ribonucleoprotein complex involving Ago2 as a direct binding partner. Alternatively, circulating miRNAs may be bound to various lipoproteins. Finally, miRNAs may be released in a form encapsulated in extracellular vesicles (EVs). Under normal circumstances, the non-membrane, Ago2-bound form of circulating miRNAs is predominant, while disease states are variably associated with EV-encapsulated circulating miRNAs [22,59]. According to the size of EVs, one can distinguish exosomes or nanoparticles with 50 to 100 nm in diameter formed during exocytosis, microparticles with 100 to 1000 nm in diameter that contain outward-oriented phosphatidylserine formed by budding/blebbing of the plasma during membrane programmed cell death, and apoptotic bodies that exceed 1000 nm in size and represent a more advanced form of cellular collapse with larger pieces included [59]. There is growing evidence that EV-packaged miRNAs in NAFLD are associated with hepatocellular injury due to lipotoxicity [60].

Several groups have studied the association of circulating miRNAs with NAFLD and most information has been collected about miR-122 in these studies. In a recent work on the association of circulating miRNAs with the severity of liver fibrosis and the development of hepatocellular carcinoma, miR-122, miR-34a and miR-16 were found to be significantly higher and positively correlated with disease severity in 34 patients with NAFLD compared to 19 healthy controls [61]. Circulating miR-122 levels were also found increased both in the exosome-rich and protein-rich serum fractions of mice with methionine-choline-deficient diet-induced NAFLD [43]. Since miR-122 levels correlated with serum alanine aminotransferase (ALT) levels, these authors concluded that increased circulating miR-122 is likely an indicator of its release from injured hepatocytes. A subsequent study on high-fat diet-induced NAFLD in rats found up to 10-fold increases in miR-122 levels in the absence of serum ALT changes suggesting that serum miR-122 levels may in fact provide a biomarker for early-stage NAFLD, at least in this experimental model [62].

In two different mouse models of dietary-induced NAFLD, proteomic and molecular analysis found a large presence of circulating miRNAs compared to controls with distinct peaks on mass spectroscopy analysis corresponding to exosomes and microparticles, both fractions being abundant in miR-122 and miR-192 [60]. In the same work, electron microscopy analysis detected a significant number of EVs located between the hepatocyte villi and sinusoidal wall, positively correlating with the severity of experimental NAFLD [60]. In addition, there is emerging evidence that miRNAs encapsulated in EVs may have an important role in cell-to-cell crosstalk and may contribute to liver disease [63]. In a very recent work, various animal models of experimental NAFLD, circulating miR-128-3p levels were markedly associated with the extent of fibrosis and depletion of miR-128-3p content of hepatocellular EVs with antagomiR-128-3p resulted in diminished stellate cell activation and down-regulation of pro-fibrotic markers [64].

In a recent effort to establish circulating miRNA signatures by global miRNA profiling in human serum and liver samples of patients with NAFLD, serum levels of miR-122, miR-192, miR-19a, miR-19b, miR-125, and miR-375 were found at least two-fold higher than in healthy controls, while liver tissue expression of these miRNAs was correspondingly lower in the NAFLD group [65]. Of note, miR-122 had the most remarkable changes, with its primarily Ago2-free levels being 7.2-fold higher in NASH *vs.* healthy controls and 3.1-fold higher in NASH *vs.* steatosis. Circulating miR-122 levels also correlated with fibrosis and predicted fibrosis severity better than cytokeratin-18, ALT, or AST, with no further improvement by combining these covariates. This study also found an association of circulating miR-192 and miR-375 levels with NAFLD severity [65].

While circulating miRNAs are attractive biomarkers, some caution may be advised. The cause-and-effect relationship between circulating miRNAs and liver disease remains incompletely understood. It is also unclear if the release of circulating miRNAs is tumor-specific or even if they are liver-originated [58,66]. Moreover, it is estimated that there are approx. 100 to 500 miRNAs circulating in the serum or plasma [24], while the number of miRNAs found in liver tissue is significantly higher, indicating simultaneous need for tissue-based research to uncover the full spectrum of biological effects played by miRNAs in the development and progression of NAFLD.

## 6. Systems Biology Approaches to Elucidate the Role of miRNAs in NAFLD

MiRNAs are involved in the regulation of all levels of complex biological organization such as signaling, metabolic, protein–protein interaction, and gene regulatory networks. A substantial degree of multi-functionality and redundancy between genes and miRNAs indicate that we may not be able to decipher the biological role of any miRNA species in isolation. Consequently, there is a great need to develop a systems-level understanding of miRNA biology. In a recent effort of applying high-throughput sequencing approaches to the analysis of miRNAs that may mediate liver fibrosis in NAFLD, global miRNA sequencing was performed in wedge liver biopsy specimens obtained from bariatric surgery of 15 cases with advanced fibrosis (F3/F4) and 15 cases with no fibrosis (F0) [67]. Differential miRNA expression analysis found that 43 miRNAs out of a total of 777 had significantly increased (*n* = 14) or decreased (*n* = 29) expression in association with advanced fibrosis. Based on the predicted gene targets of these deregulated miRNAs, functional enrichment analysis identified 110 molecular pathways including apoptosis, transforming growth factor-beta (TGFβ) signaling, fibrosis and stellate cell activation, regulation of endothelial-mesenchymal transition, IGF-1 and insulin signaling, extracellular signal regulated kinase (ERK)/MAPK pathway, and cholestasis, which are potentially associated with NAFLD pathogenesis [67]. Surprisingly, advanced liver fibrosis was not associated with differential expression of miR-122 or miR-34a in this mouse model, which is at variance with prior observations and may result from species differences.

By using a twin-study design, Zarrinpar and coworkers recently examined the role of miRNAs in discordancy between twins with and without NAFLD in a cross-sectional analysis of a cohort of 40 twin pairs [68]. Six twin pairs were discordant for the presence of NAFLD and the authors identified 10 miRNAs corresponding with this discordance. In their comparison, miR-331-3p and miR-30c were the most discriminative and were also identified among the 21 miRNAs differentiating NAFLD from non-NAFLD twins in the entire twin cohort. Moreover, heritability analysis found miR-331-3p and miR-30c to be highly heritable. Targeted interactome analysis utilizing Kyoto Encyclopedia of Genes and Genomes (KEGG) pathways for cancer and lipid metabolism revealed that common predicted gene targets of miR-331-3p and miR-30c are highly connected. Interestingly, miR-122 and miR-34a* in this analysis were primarily associated with non-shared environmental factors, suggesting that these miRNAs may only gain a prominent role later in the course of NAFLD through external perturbations [68].

Utility of systems biology approaches based on published experimental data was demonstrated by an *in silico* study in which free-text co-occurrences of genes and proteins in PubMed abstracts were used to create functional molecular maps for disease pathways shared between NAFLD and alcoholic fatty liver disease (AFLD) [69]. Besides other important conclusions on common and unique mechanisms of the pathogenesis, integrative functional analysis based on predicted gene targets recognized several miRNAs with potential effects in AFLD, NAFLD, or both. For instance, the analysis placed miR-7a and miR-199a-3p in the shared area with overlapping roles in apoptosis and inflammation pathways [69].

## 7. MiRNAs as Therapeutic Targets in NAFLD

The complex role that miRNAs play in the regulation of gene expression makes these molecules highly attractive therapeutic targets. Since miRNAs generally act by repressing gene transcription, the goal is either to negate the inhibitory effect of miRNA by silencing or to restore inhibition of the target genes by supplanting the sense miRNA. A number of approaches have been tested for miRNA silencing. Antisense oligonucleotides (antagomirs) with complementarity to the mature miRNA strand may be chemically modified to confer nuclease resistance, increase binding affinity, and reduce biological toxicity [23]. A stoichiometrically more efficient strategy is to use miRNA sponges that contain several tandem-binding sites to the miRNA of interest [70]. Competent endogenous RNAs (ceRNAs), such as circular RNAs, long non-coding RNA (lncRNAs), or so-called pseudo-genes may suppress miRNA action by competing with mRNAs [23]. Additional methods of miRNA inhibition involve miRNA “erasers” that are deployed by the help of viral vectors and miRNA decoys that bind as a regular miRNA to the target but without any effect on translation [26,71]. Yet another technique employs locked nucleic acid chemistry to construct inaccessible RNA molecules with modified ribose moieties, which have very short sequences and carry the risk of nonspecific and broad action due to full complementarity [22].

Promoting tumor suppression and blocking oncogenic pathways through the modulation of miRNAs are examples for reaching similar therapeutic effects through opposing approaches. Exosome-mediated delivery of let-7 miRNAs to restore inhibition of the epidermal growth factor receptor in cancer cells (a method named “exocure” by the authors) has proved to be a promising approach [72]. By contrast, aberrant upregulation of oncomirs such as miR-21 may result in insufficient tumor suppression or apoptotic cell death as originally demonstrated in the case of miR-21 in glioblastoma, making therefore miRNA inhibition a desirable goal [73]. Inhibition of miR-21 by antisense oligonucleotides is associated with deregulation of multiple growth-promoting pathways resulting in loss of cell migration, suppression of clonogenic growth, and induction of apoptosis in most HCC cells lines tested in a recent multicenter study [74]. Dependency of HCC growth on miR-21 was also demonstrated in a xenograft model, adding support to miR-21 inhibition as a promising therapeutic intervention [74].

Efforts to use these technologies in the treatment of NAFLD are only beginning. Importantly, delivery of miRNA targets packed into liposomes or other lipophilic nanoparticles to the liver is quite efficient through the portal circulation with the first-pass effect [75]. Miravirsen, a locked nucleic acid-modified antagomir developed to inhibit miR-122 was the first parenterally administered miRNA drug developed against HCV with a potential impact on NAFLD since miR-122 has many predicted gene targets involved in lipid metabolism [76]. It is a potential concern that miR-122 is downregulated in HCC and its role in hepatocarcinogenesis remains to be elucidated [25]. In general, therapeutic approaches based on miRNA targeting may be problematic due to the high-level redundancy and multi-functionality of this gene regulatory system, which makes it potentially difficult to deliver specific miRNA effects without the risk of collateral damage. Use of genome editing technologies such as the novel and versatile clustered regularly interspaced short palindromic repeats (CRISPR)-associated protein-9 systems will likely allow for the increased use of sequence-specific miRNA inhibition [77]. Further studies will be necessary to explore the value and safety of this and other miRNA modulators in the therapy of NAFLD.

## 8. Perspectives

Regulation of gene expression by miRNAs has become one of the most dynamically growing fields in biomedical research. Differential miRNA expression by microarray analysis and more recently by next-generation sequencing continues to be easier, cheaper, and more comprehensive due to methodical advances. Circulating miRNAs are likely to become more informative and reliable biomarkers. Approaches to mimic or inhibit miRNAs for therapeutic purposes will be increasingly refined to prevent nonspecific effects. Data are emerging in many different areas and liver diseases are no exception. We may say with sufficient optimism that our understanding of the pathogenesis, capacity to prognosticate, and ability to treat NAFLD will be greatly affected by these developments.
